# Digital Health Interventions in Prevention, Relapse, and Therapy of Mild and Moderate Depression: Scoping Review

**DOI:** 10.2196/26268

**Published:** 2021-04-16

**Authors:** Pinar Tokgöz, Robert Hrynyschyn, Jessica Hafner, Simone Schönfeld, Christoph Dockweiler

**Affiliations:** 1 School of Public Health Bielefeld University Bielefeld Germany; 2 Charité – Universitätsmedizin Berlin Corporate member of Freie Universität Berlin and Humboldt-Universität zu Berlin Institute of Health and Nursing Science Berlin Germany; 3 LWL-Klinik Lippstadt und Warstein Lippstadt Germany; 4 Universität Witten/Herdecke Institut für Integrative Gesundheitsversorgung und Gesundheitsförderung Witten Germany

**Keywords:** digital health, depression, scoping review, health care

## Abstract

**Background:**

Depression is a major cause for disability worldwide, and digital health interventions are expected to be an augmentative and effective treatment. According to the fast-growing field of information and communication technologies and its dissemination, there is a need for mapping the technological landscape and its benefits for users.

**Objective:**

The purpose of this scoping review was to give an overview of the digital health interventions used for depression. The main goal of this review was to provide a comprehensive review of the system landscape and its technological state and functions, as well as its evidence and benefits for users.

**Methods:**

A scoping review was conducted to provide a comprehensive overview of the field of digital health interventions for the treatment of depression. PubMed, PSYNDEX, and the Cochrane Library were searched by two independent researchers in October 2020 to identify relevant publications of the last 10 years, which were examined using the inclusion and exclusion criteria. To conduct the review, we used Rayyan, a freely available web tool.

**Results:**

In total, 65 studies were included in the qualitative synthesis. After categorizing the studies into the areas of prevention, early detection, therapy, and relapse prevention, we found dominant numbers of studies in the area of therapy (n=52). There was only one study for prevention, 5 studies for early detection, and 7 studies for relapse prevention. The most dominant therapy approaches were cognitive behavioral therapy, acceptance and commitment therapy, and problem-solving therapy. Most of the studies revealed significant effects of digital health interventions when cognitive behavioral therapy was applied. Cognitive behavioral therapy as the most dominant form was often provided through web-based systems. Combined interventions consisting of web-based and smartphone-based approaches are increasingly found.

**Conclusions:**

Digital health interventions for treating depression are quite comprehensive. There are different interventions focusing on different fields of care. While most interventions can be beneficial to achieve a better depression treatment, it can be difficult to determine which approaches are suitable. Cognitive behavioral therapy through digital health interventions has shown good effects in the treatment of depression, but treatment for depression still stays very individualistic.

## Introduction

Depressive disorders are among the most significant and widespread diseases, and their relevance will continue to increase in the coming years. Nearly 300 million people are affected worldwide [1]. Depressive disorders have a high risk of chronicity and are associated with a substantially increased probability of developing further comorbidities with corresponding effects on the quality of life of those affected. Psychiatric care is facing different structural problems of care, with underuse and misuse of care that is evident in practice [1]. Barriers to access to psychiatric specialists or psychotherapeutic care, lack of cross-sectoral and interdisciplinary care, and long waiting lists are also challenging. It is estimated that less than 50% of those currently affected receive therapy that is appropriate to their needs and requirements according to current scientific criteria. Although there is an increasing awareness of mental health issues, accessibility to health care has been a key problem [2].

Against this background, psychological, psychotherapeutic, and psychiatric care are experiencing remarkable developments in technology-supported care concepts. A broad spectrum of digital health interventions (DHIs) in psychiatric care already exist today [3]. DHIs enable new forms of interaction and knowledge-based reproduction in the field of health. The constantly growing number of interventions extends from outpatient digital health care programs to telephone, video, or text-based interactions with the therapist and complex online-based intervention programs. Unlike face-to-face treatment, such support systems are easily accessible and standardized, and they can reduce the fear of stigmatization, as they can be used in private and at the convenience of the patient [3].

A steadily increasing amount of empirical data show first indications of patient-related benefits of DHIs, especially the reduction of depressive symptoms; improvement in quality of life; and reduction of direct, indirect, and intangible costs [3]. These potential benefits could be effective for patients and the health system if successful acceptance of DHIs is achieved [4].

This scoping review is part of a research project that examines the multiperspective and participatory development of technology-supported care for people with depressive disorders. The purpose of this scoping review was to give an overview of DHIs used in different fields of depression care. The main goal was to provide a comprehensive review of the system landscape and its technological state and functions, as well as the evidence and benefits for users.

To this end, the following research questions have been addressed:

- What types of DHIs for the treatment of mild and moderate depression have been developed, and how can the functions be described?
- How can the benefits of DHIs in the care of mild and moderate depression be described?

## Methods

### Overview

A scoping review has been conducted to identify knowledge gaps, set research agendas, and identify implications for intervention development. Although scoping reviews are related to systematic reviews, they differ in numerous ways. Scoping reviews present a broader overview of evidence pertaining to a specific topic, irrespective of study quality [5], useful when emerging topics are discussed to clarify key concepts and research gaps. Systematic reviews focus more on specific research questions with a priori defined criteria. Therefore, scoping reviews generate hypotheses, while systematic reviews focus more on testing hypotheses [6]. Results were reported according to the Preferred Reporting Items for Systematic Reviews and Meta-analyses (PRISMA) guidelines of systematic reviews because there are no reporting guidelines for scoping reviews. Research questions and inclusion criteria were adapted from Arksey and O’Malley [5].

### Inclusion and Exclusion Criteria

The following criteria have been developed in coordination with the partners of the research project. The inclusion criteria comprised (1) all DHIs for the treatment of depression, (2) quantitative study design, (3) language: English or German, and (4) published between 2010 and 2020 since the technological development of DHI is dynamic. Exclusion criteria were (1) study participants aged younger than 18 years, (2) no diagnosis of depression, (3) severe course of illness, (4) psychotic symptoms, (5) concurrent medication therapy, or (6) DHIs with no clear relation to depression treatment.

### Literature Search

The search was conducted in October 2020 in the databases PubMed, PSYNDEX, and the Cochrane Library. The following search strategy was used: (depression OR depressive disorder OR depressive episode) AND (online based* OR mobile* OR ehealth* OR “electronic mental health” OR ”e-mental health” OR online-based* OR internet-based* OR web-based* OR computer-based).

### Data Extraction

The articles were extracted using standardized table formats. To provide an overview of the aspects of DHIs for depression considered here, the following taxonomy is presented in the tables:

- Authors, year, country, and funding of the study
- Study design
- Study period
- Sample size information
- Technology description and functions
- Relevant outcomes and effects

Moreover, the results were analyzed regarding the effects of the reduction of depressive symptoms and specific characteristics of the DHI. For this purpose, contingency tables were used to show frequency distributions between the benefit and the technology used and the benefit and the therapy form used. Furthermore, graphs were built to show which functions have been used by the different therapy forms.

## Results

### Study Selection

**Figure 1:**
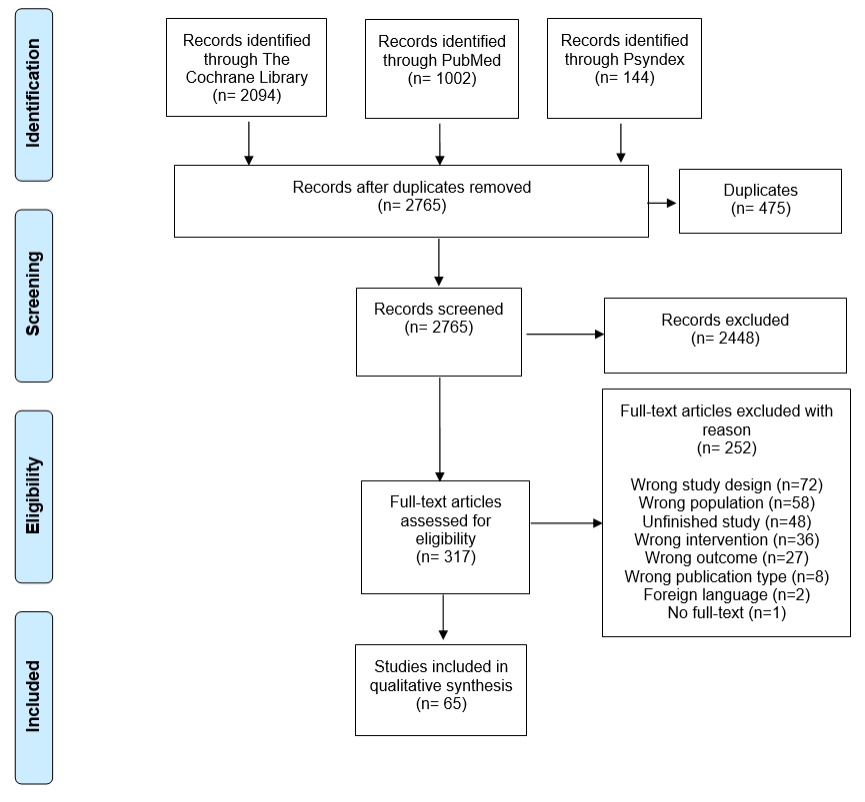
PRISMA flow diagram of literature search and selection process [7].

In total, 3040 publications were identified. These publications were transmitted to Rayyan, which is a free web tool designed to help researchers conducting systematic reviews. Using this program, the 2 independent researchers (PT and RH) screened the articles according to inclusion and exclusion criteria. In case of disagreement, consensus has been made by the opinion of a third researcher (JH). A total of 65 studies were included in the qualitative synthesis (Figure 1).

**Figure 2:**
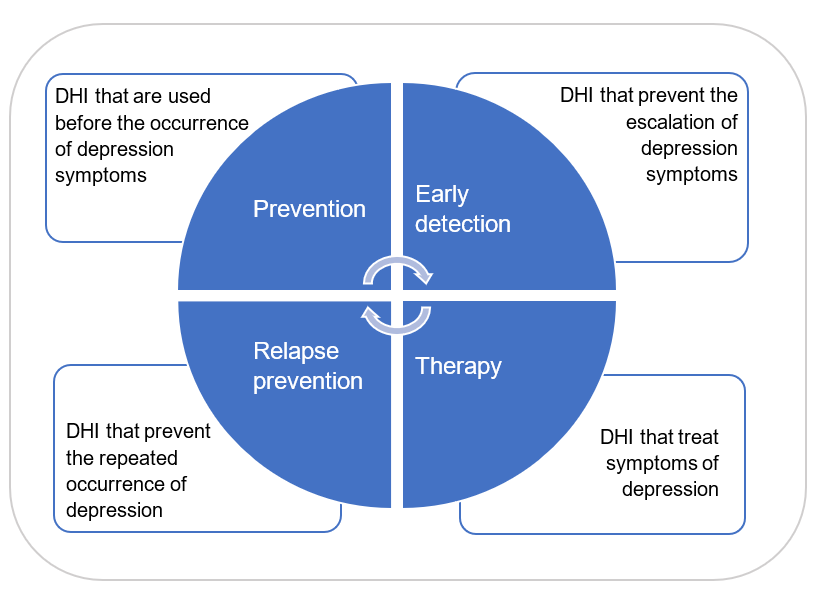
Classification of digital health intervention application areas.

In a first step, the field of depression care was categorized in 4 application areas: prevention (1 study identified), early detection (5 studies), therapy (52 studies), and relapse prevention (7 studies; Figure 2). In a further step, this standardization enabled technology mapping and a detailed description of the technologies and their benefits.

### Data Description: Prevention/Early Detection

In the area of prevention, only one study was available in context of this scoping review [8]. The study was conducted in Germany and funded by the European Union (EU). Its duration was 6 weeks, with follow-ups after 6 months and 12 months. The study population consists of people with underlying depressive symptoms. Differentiation between an intervention group (IG) and control group (CG) was made (IG=202 and CG=204; Multimedia Appendix 1).

The intervention GET.ON Mood Enhancer Prevention is a web-based guided self-help intervention based on psychoeducation, problem-solving therapy, and behavioral activation. The concepts were conveyed via multimodal and interactive elements in 6 sessions of about 30 minutes each with individual feedback by an online trainer. The CG received psychoeducation via the same web-based platform, but without the guidance of an online trainer. The primary outcome of the study was the diagnosis of major depression, which was recorded using the *Diagnostic and Statistical Manual of Mental Disorders, Fifth Edition* (DSM-5) criteria and a Structured Clinical Interview for DSM-5 (SCID).

Five studies were identified in the area of early detection (Multimedia Appendix 1). Two of the studies were conducted in Germany [9,10], 2 in the United States [11,12], and 1 in Romania [13]. Two studies were financed by the EU [9,10], and 3 were funded by countries [11-13].

In all 5 studies, the study populations consisted of people with underlying depressive symptoms (ie, no manifest depressive disorder). In 4 studies, a differentiation was made between the IG and CG. The number of participants ranged from 35 to 202 in the IG (mean 102; mode 85.5) and from 36 to 204 participants in the CG (mean 93.7; mode 69). One study was a comparison of different interventions and did not have a CG [12]. The study periods ranged from 3 to 24 weeks.

Three out of the 5 studies chose a mixed form as treatment approach. In 2 studies, the intervention consisted of web-based cognitive behavioral therapy (CBT) and problem-solving therapy [9,10]. One study consisted of an app-based intervention based on CBT and problem-solving therapy, as well as an information control app [12]. In all 3 studies, the participants of the IG received support or reminder from an online trainer via a news system. Optionally, the participants could also choose to receive motivational text messages regularly to ensure continuity. In Place et al [11], the intervention consisted of a monitoring system that collected different types of metadata and clinicians providing feedback. In Buntrock et al [9], the CG only received psychoeducation through the web-based platform. In 2 studies, participants of the CG had unlimited access to treatment as usual (TAU) during the study period and were given access to the web-based intervention after the study period [10,11].

The study by Tulbure et al [13] investigated a web-based transdiagnostic intervention. Thereby, therapy concepts of different mental diseases were transferred. In this study, concepts from the field of anxiety disorders were projected onto the field of depressive disorders. The IG received a web-based multimodal intervention in which additional writing tasks were completed. The participants could perform the intervention on a computer, tablet, or smartphone. Trained personnel monitored the activity of the participants. In addition, there was personalized feedback on writing tasks. Furthermore, participants received a reminder if inactivity was detected. No information was provided regarding the therapeutic approach. The CG received reading access to the platform [13].

All studies described depression-specific symptoms as primary outcome [9-13]. Additionally, in 2 studies, adherence to the intervention [9,13] was indicated; in 1 study, quality of life [13] was.

### Data Description: Relapse Prevention

In the area of relapse prevention, 4 studies from Germany [14-17], 1 study from the Netherlands [18], 1 study from Denmark [19], and 1 from the United States [20] were identified (Multimedia Appendix 1).

The study periods ranged from 4 weeks to 104 weeks (24 months). Four studies were financed by countries [14,17,18,20], and 2 studies were privately financed [16,19]. One study did not provide information on financing [15]. Six studies differentiated between an IG and a CG [14-18,20]. The studies revealed large differences in sample size, ranging from 41 to 264 participants (mean 182; mode 217.5) when the IG and CG were combined. The sample sizes in the experimental arms of the studies varied between 21 and 230 participants (mean 115.2; mode 120). In the CG, the samples varied between 20 and 230 participants (mean 101.2; mode 91.5).

The interventions showed differences in their implementation. While almost all of them used a feedback function to indicate therapy success or remind patients to perform tasks, the therapy strategy differed. Four out of the 7 studies chose CBT as a therapeutic approach [16-18,20]. Some studies used CBT, either through a telerehabilitation program [16], mobile app [18], or web-based program [20]. Kraft et al [14] opted for a mindfulness-based exercise in which patients received feedback on the success via SMS. Lauritsen et al [19] used a web-based self-assessment tool that monitored participant mood, sleep, and activity, and Zwerenz et al [17] used a web-based self-help program based on CBT. One study did not provide any information on the chosen therapeutic approach [15]. Instead of the technical intervention, the CG of all studies received the nontechnical comparative therapy (ie, a comparable therapy) performed without the use of technical aid or TAU.

Five studies described depression-specific symptoms as primary outcome [15-17,19,20]. Additionally, adherence to the intervention [14,19] and quality of life [17,19] were indicated.

### Data Description: Therapy

For the area of therapy, 52 studies were identified. A total of 12 studies were conducted in the United States [21-32], 6 studies in the Netherlands [33-38], and 11 in Germany [39-49]. Four studies were conducted in Australia [50-53]. Three studies were conducted in Switzerland [54-56] and 4 in Spain [57-60]. In Canada [61,62], Finland [63,64], Sweden [65,66], and Great Britain [67,68] 2 studies each were identified. New Zealand [69], Austria [70], Ireland [71], and Japan [72] were each represented with 1 study (Multimedia Appendix 1).

Of those, 27 were sponsored by countries [23,25,26,28-32,35,40,41,43,44,46,51,52,57-62,65,66,68,69,71] and 11 studies were privately financed [24,33,34,36,37,48,49,53,55,56,67]. The EU funded 2 studies [39,42]. One study indicated a mixture of private and state funding [21]. Eleven studies did not provide any information of financial sources [22,27,38,45,47,50,54,63,64,70,72].

In the studies differentiating between an IG and CG, the number of participants ranged from 10 to 1904 participants in the IG (mean 191.0; mode 88) and from 8 to 1901 participants in the CG (mean 179.1; mode 67.5). The intervention periods of the studies ranged from 2 to 52 weeks (mean 11 weeks).

The majority of studies (41/52) differentiated between an IG and CG and were conducted as randomized controlled trials (RCTs) [22,24,27-29,33-52,54,55,57-61,63-69,71,72]. Seven studies were conducted as pilot studies [25,26,30-32,56,62]. Two studies had a quasi-experimental design [23,70]. One study was conducted as a usability study [53] and 1 as a controlled clinical trial [21].

### Technology Mapping: Therapy

The therapy area yielded the largest number of studies. Therefore, the following technology mapping and analysis focus on therapeutic interventions for the reduction of depressive symptoms. In terms of therapeutic approach, CBT, acceptance and commitment therapy (ACT), and problem-solving therapy was considered because those are the most used approaches, and there was sufficient evidence to analyze. Studies were examined according to the type of technology used, medium used, and functions offered. Cross-references between the levels were drawn with the help of cross tables.

CBT was the dominant form of therapy in 54% (28/52) of the studies [21,23,24,28,30,32,39-41,43-46,48-50,52,55-57,60-62, 65-67,70-72]. In 19% (9/52), mixed forms were used, consisting of 2 or more forms of therapy [27,31,33,34,42,47,51,58,59]. Other therapy approaches such as ACT (5/52, 9%) [26,35,38,63,64] and problem-solving therapy (4/52, 8%) [29,36,37,69] were significantly less common in interventions. The remaining studies either focused on cognitive restructuring [54], cognitive control therapy [22], behavioral activation [45,68], or supplied no information about the therapeutic approach that was used [25].

Considering how therapy was technologically implemented, differences become apparent. Most of the studies (39/52, 75%) used a web-based system in which an application was used online [21-23,28,33-46,48,49,52-55,57-67,69-72]. This form of therapy provision was the most common form. Mixed forms were also frequently used (5/52, 11%) using web-based and smartphone-based approaches [24,27,30,32,68]. Smartphone-based approaches were used by 12% (6/52) of studies [25,26,31,47,50,56]. One study (2%) featuring problem-solving therapy used virtual reality for delivery, which entailed a virtual therapist giving instructions on skills in problem solving [29].

In addition, we analyzed which functions were integrated in the DHI. Multiple answers were possible, depending on which functions were given. Most of the DHIs (33/52, 63%) were offered as guided interventions in which a therapist supported the participants [21,25,26,28,29,31-39,42-44,47,49,54,55,57-60,62-64,67-71], with 15% (11/52) conducted as unguided interventions [22,23,27,30,40,45,46,49,50,52,61] and 15% (11/52) using the reminder function to encourage patients to perform daily exercises or self-assessments [24,32,41,47,49,51,63-65,67,68]. While some studies (6/52, 8%) used tracking functions with biosensors [24,50,51,53,56,59], 3% (2/52) used diary functions [50,51] and 3% (2/52) used discussion forums [32,62]. A total of 9% (5/52) of studies used educational elements for the treatment of depression [53,56,66,70,72].

### Evidence of DHI in Therapy for Depression

A total of 71% (37/52) of studies reported improvements in depressive symptoms [22,30,32,37,40,42,44,46,49-51,53-57,59-64,67,68,70-72], 27% (14/52) reported no (significant) effects [21,31,33-36,41,45,47,48,58,65,66,69], and 2% (1/52) did not consider depressive symptoms as an outcome [52].

**Figure 3:**
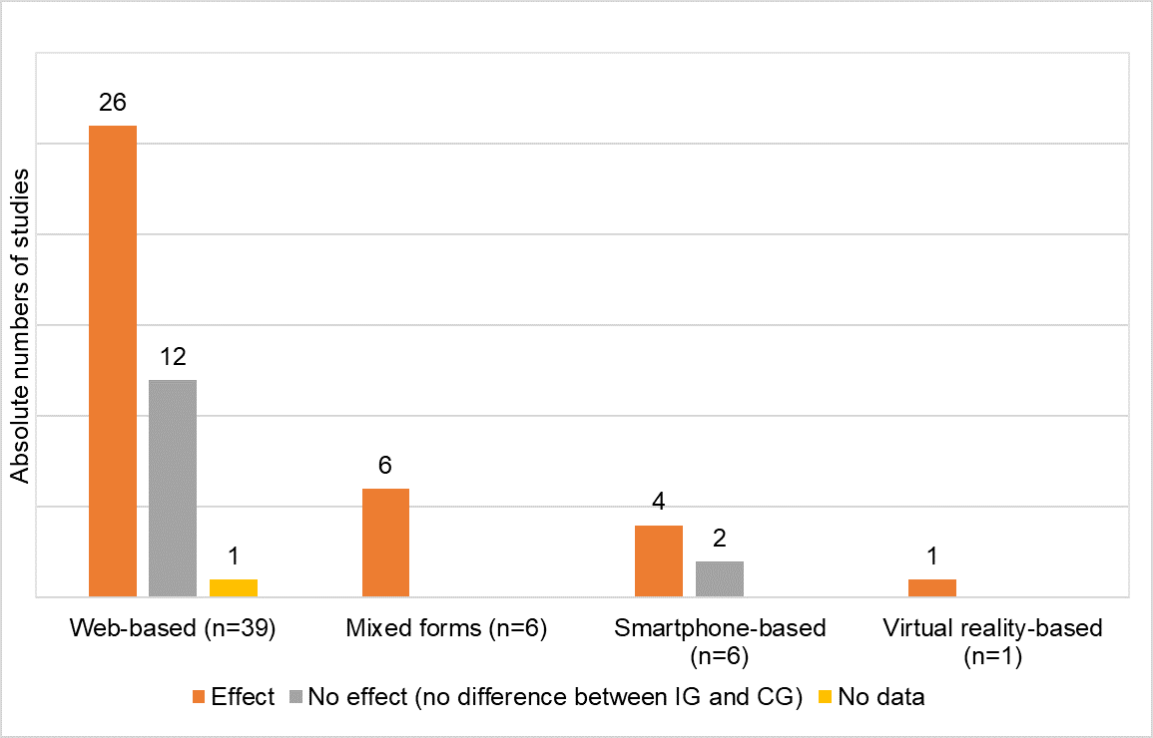
Evidence for digital health interventions classified by technology.

In Figure 3, the type of technology used with reported benefits of the studies is shown. In the case of web-based provision of DHIs, improvements of depressive symptoms becomes clear. A total of 67% (26/39) of studies identified a benefit [22,23,28,37,40,42,44,46,49,52,55,57,59-64,67,70-72], 31% (12/39) did not verify an improvement in depressive symptoms [21,33-36,41,45,48,58,65,66,69], and 1 study did not provide results for depressive outcomes [52]. All studies that used mixed forms of technology demonstrated a benefit. However, due to the small number of studies, this should be interpreted cautiously.

### Evidence for CBT

**Figure 4:**
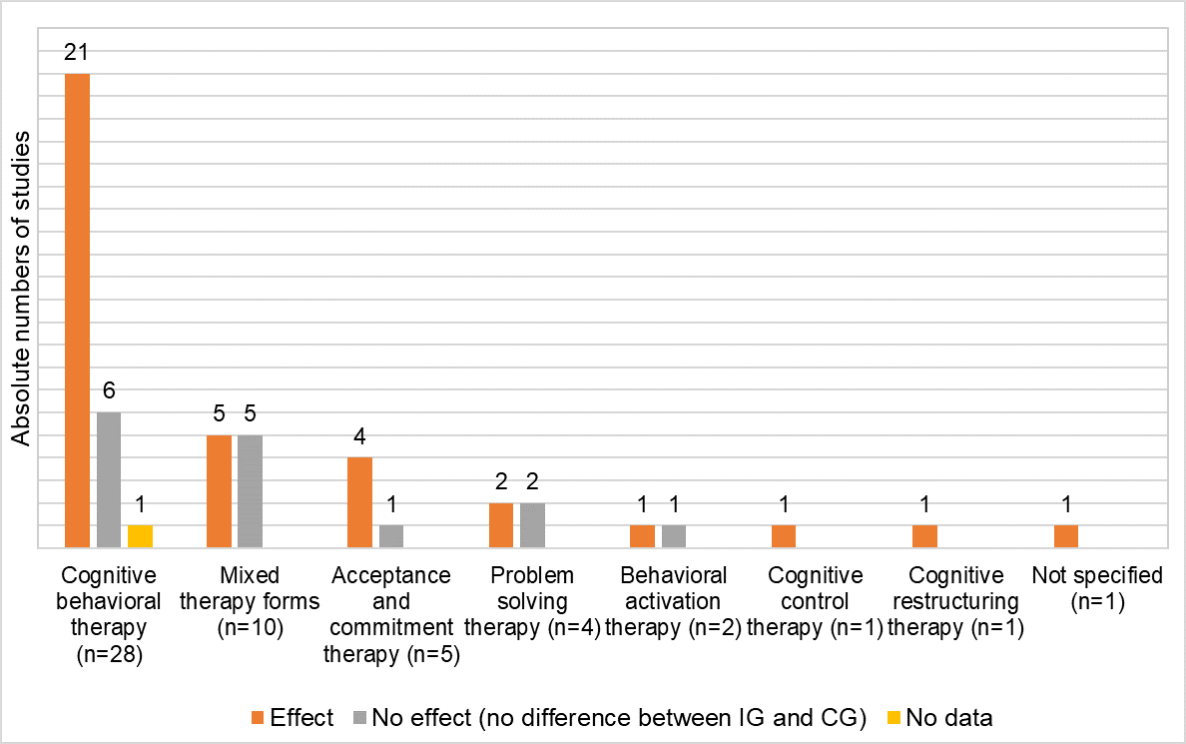
Evidence of digital health interventions classified by therapeutic approach.

In Figure 4, the therapy approach used with reported benefits is shown. A total of 75% (21/28) of studies that chose a CBT approach reported a positive effect on the reduction of depressive symptoms [23,24,28,30,32,39,40,44,46,49,50,55,57,60,62,67,70-72], 21% (6/28) could not find any effect in terms of reduction [21,41,43,47,65,66], and 4% (1/28) did not report any information on the depression outcome [52].

Of the studies with a positive effect, 76% (16/21) were transmitted via web-based applications [23,28,39,40,44,46,49,55,57,60-62,67,70-72], 48% (10/21) were designed as guided [28,39,44,55,57,60,62,67,70,71] and 29% (6/21) as unguided interventions [23,40,46,49,61,72].

In contrast, 6 studies could be identified that showed no or neutral results: 5 were web-based interventions [21,41,43,65,66] and 1 study was designed as an app [47].

### Evidence for Problem-Solving Therapy

The evidence for benefits using the problem-solving therapy approach is controversial. Of the studies identified, 50% (2/4) determined a benefit in terms of an improvement in depressive symptoms [29,37] and 50% (2/4) could not determine a difference between the IG and CG or simply do not represent significant improvement in the IG [36,69]. One of the studies with a reported benefit was a web-based application [37] and the second was a virtual reality intervention [29].

### Evidence for ACT

Of the studies that chose ACT as a therapeutic approach, 80% (4/5) demonstrated a benefit regarding depressive symptoms [26,38,63,64]. One of the studies with a reported benefit was an app-based intervention [26] and 3 were designed as guided web-based interventions [38,63]. The study that did not indicate a benefit was a web-based intervention with automated feedback function [35].

## Discussion

### Principal Findings

The number of DHIs for treating depression is quite comprehensive. Focusing solely on therapeutic setting is not sufficient to show the different approaches of depression care. However, most of the studies were found in the therapy area. Of the 65 studies included, 52 focused on a therapy setting, 7 on relapse prevention, 5 on early detection, and 1 on the prevention of depression.

The most dominant approaches were CBT, ACT, and problem-solving therapy. Regarding the efficacy, most of the studies finding a benefit regarding symptom severity used CBT. Most studies found a significant effect of digital CBT for depression symptoms. Other studies not using DHIs for the provision of CBT have similar results for the efficacy of CBT regarding reduction of depression symptoms. Especially for mild and moderate depression, there is good evidence for symptom reduction [73]. Beyond that, the DHIs in most of the identified studies were realized through web-based technologies; mobile or smartphone-based interventions were still underrepresented.

Most of the included studies compared participants treated with DHIs with those not treated at all (eg, waitlist). In contrast, there were only a few studies that compared the efficacy of DHIs with traditional psychotherapy. It is not surprising that DHIs work better than no treatment at all. However, their efficacy is quite remarkable. Additionally, most studies were conducted under ideal controlled conditions, and studies investigating effectiveness in everyday life are still missing. For this reason, there are emerging discussions about adaptive study designs such as n-of-1 or interrupted time-series designs, which can be better suited to evaluate DHIs. Although decision makers or payers still prefer RCTs as the gold standard for evidence, there are limitations when it comes to the measurement of efficacy and effectiveness for DHIs. Although health insurance companies offer their insureds DHIs, only 3% to 25% of patients take advantage of them [74]. Reasons for this are low expectations of their effectiveness, concerns about data security, poor user-friendliness, general skepticism about psychotherapy, and little experience with the internet in general [74].

### Comparison With Prior Work

However, even for CBT, which is increasingly delivered by computerized forms, there is good evidence supporting the stated effects in this study. In the meta-analysis of Andersson and Cuijpers [75] that examined the effects of computerized CBT compared with face-to-face interventions, the authors found that computerized treatments do have promising potential for treating depression, and the computerized treatments were statistically significantly superior (posttest effect size: 0.41, 95% CI 0.29-0.54, *I*^2^=57%) to face-to-face interventions.

The dissemination of technological systems poses a challenge for the field. DHIs developed within a research context vary in their implementation and technological approaches. Differences in technological attributes have been discussed in the literature. Studies have consistently demonstrated that effects appear to increase with higher levels of human guidance (eg, no guidance vs administrative guidance vs therapist guidance) [4,76]. Johansson et al [77] showed in a systematic review of 25 controlled trials that guided internet-based CBT is more effective than unguided internet-based CBT. Whereas the effect sizes in studies with people who had no therapist contact either before or during the treatment were relatively low (average effect size *d*=0.21), they improved with more support from the therapist. Studies with people who had contact with a therapist before and during the treatment reported a much higher average effect size (*d*=0.76). Wright et al [78] showed similar results in a recent study from 2019. In a systematic review with 40 RCTs, they showed that studies providing support by a therapist or clinician yielded larger effects (g=0.67) than studies with unguided interventions (g=0.24). One reason for this could be the missing therapeutic alliance was not compensated for with more content or technological support, apart from reminders. Therefore, it is possible that guidance becomes important, especially when systems are not very responsive.

Aside from this, there are many opportunities for DHIs in the treatment of depression. In fact, interventions are mostly delivered on the internet. Only a few publications considering mobile interventions were identified. This is surprising since smartphones are ubiquitous and highly prevalent nowadays. A review from 2015 identified 82 mobile apps for depression treatment [79]. Another review found that only 5 of those apps had been empirically evaluated in RCTs [80]. Regarding the high diffusion of commercially driven apps, scientifically evaluated apps are needed in future. In addition, innovative technologies using social media or virtual reality are underrepresented in this review.

### Limitations

Most of the studies were found in the area of therapy. One reason for this could be that preventive offers are not recognized the way therapeutic interventions are, even if those preventive interventions are advisable due to individual risk factors. It could be helpful to provide a wider range of preventive services and a larger number of psychotherapists as well as general practitioners to give their patients recommendations for preventive interventions.

All studies tried to determine the effects of depression-specific symptoms, but in doing so the authors of the studies used different instruments. While some researchers used the Center for Epidemiological Studies Depression Scale or the Quick Inventory of Depressive Symptomatology–16 item scale, the Patient Health Questionnaire–9 Item and Beck Depression Inventory II scales were most commonly used. Because of this heterogeneity, there is a limitation considering the comparability of studies.

A limitation concerns the scope of the reviewed publications. Systems developed for children and adolescents and women with postpartum depression and studies published before 2010 were excluded. Therefore, it is necessary to investigate in further research if the findings can be generalized to those types of systems. As data security is an important concern of DHIs, consistent guidelines and criteria for future systems are needed. Despite these limitations, the findings of our analysis highlight some of the challenges and opportunities for the use of DHIs for depression.

### Conclusion

The aim of this scoping review was to approach the state of DHIs for mild and moderate depression and try to describe those interventions for depression care. It is known that DHIs have great potential to complement the treatment of depressive people and intensify traditional psychotherapy. This review indicates DHIs also show great potential in avoiding depression (prevention and early detection) and preventing relapse, which is currently underrepresented in the literature. The field of DHIs is constantly increasing, and there is no general evidence of the effect of these interventions, but most of the studies included in this review improved the effects of DHIs for the reduction of depressive symptoms. Furthermore, results showed that most of the DHIs provided web-based CBT and a combination of several technological options (eg, reminder, tracking). Even with automated programs and contact with psychotherapists (eg, contact through chats or email), people show great improvement in their symptoms in comparison to interventions without support at all. It appears to be crucial for people to have professional guidance. Further research is needed to investigate whether more technologically advanced systems lead to higher adherence and effects for the reduction of depressive symptoms in addition to investigating real-world effectiveness and barriers for implementation of DHIs.

## Appendix

Multimedia Appendix 1 Study characteristics of the included studies.

## Abbreviations

ACT: acceptance and commitment therapy
CBT: cognitive behavioral therapy
CG: control group
DHI: digital health intervention
DSM-5: Diagnostic and Statistical Manual of Mental Disorders, Fifth Edition
EU: European Union
IG: intervention group
LZG.NRW: Landeszentrum Gesundheit North Rhine-Westphalia
PRISMA: Preferred Reporting Items for Systematic Reviews and Meta-analyses
RCT: randomized controlled trial
SCID: Structured Clinical Interview for DSM-5
TAU: treatment as usual
