# Supplementary material for: Digital Health Interventions in Prevention, Relapse, and Therapy of Mild and Moderate Depression: Scoping Review
Source: JMIR Ment Health. 2021 Apr 16;8(4):e26268. doi: 10.2196/26268 (PMC8087966; doi:10.2196/26268)
Supplement: Multimedia Appendix 1 [file mental_v8i4e26268_app1.docx]

Table 1: Study characteristics in the area of prevention

| **Study** | **Country** | **Funding** | **Study design** | **Study period** | **Technological approach** | **Sample size** | **Outcomes**  **Depressive symptoms (A)**  **Adherence (B)**  **Quality of life (C)**  **Economic aspects (D)** | **Evidence**  **+ benefit**  **-/+ no benefit**  **~ inconsistent effects** |
| --- | --- | --- | --- | --- | --- | --- | --- | --- |
| Buntrock et al. (2016)  [8] | GER | EU | RCT | 6 weeks | IG: web-based guided self-help intervention based on psychoeducation, PST and behavioral activation (BA)  🡪personalized feedback after each unit to ensure continuity  CG: web-based psychoeducation | IG: 202  CG: 204 | A, D | A= +  D= -/+ |
| EU: European Union, RCT: Randomized controlled trial, IG: Intervention group, CG: Control group, BA: Behavioral activation, PST: Problem-solving therapy. | | | | | | | | |

Table 2: Study characteristics in the area of early detection

| **Study** | **Country** | **Funding** | **Study design** | **Study period** | **Technological approach** | **Sample size** | **Outcomes**  **Depressive symptoms (A)**  **Adherence (B)**  **Quality of life (C)**  **Economic aspects (D)** | **Evidence**  **+ benefit**  **-/+ no benefit**  **~ inconsistent effects** |
| --- | --- | --- | --- | --- | --- | --- | --- | --- |
| Buntrock et al. (2015)  [9] | GER | EU | RCT | 3-6 weeks | IG: interactive intervention  🡪optionally standardized text messages for motivation  🡪continuous guidance by "trainers" through a message system  CG: web-based psychoeducation | IG: 202  CG: 204 | A, B | A= +  B= + |
| Ebert et al.  (2017)  [10] | GER | EU | RCT | 7 weeks | IG: interactive web-based intervention  🡪optionally standardized text messages for motivation  🡪guidance by trainer to ensure continuity and feedback  CG: Access to web-based intervention 3 months after randomization | IG: 102  CG: 102 | A | A= + |
| Place et al.  (2020)  [11] | USA | State | RCT | 24 weeks | IG: app-based intervention for symptom monitoring  🡪different types of metadata are collected (call logs, geolocation data, audio recordings) for measure of depression mood  🡪visual feedback was provided to patients via app and to clinicians via desktop dashboard  🡪dashboards were reviewed in routine clinical visits  CG: TAU | IG: 35  CG: 33 | A | A= + |
| Pratap et al.  (2018)  [12] | USA | State | 3-armed RCT | 4 weeks | IG1: app-based and video-game-inspired intervention based on CBT  IG2: app-based and internet-based PST  IG3: information control app, that provides daily health tips for overcoming depressive mood  🡪each intervention app was equipped with daily reminders to enable continuity | IG1: 83  IG2: 112  IG3: 79 | A | A= -/+ |
| Tulbure et al.  (2018)  [13] | ROU | State | RCT | 10 weeks | IG: web-based multimodal intervention  🡪trained staff guides and monitors the activity of the participants  🡪personalized homework+ feedback  🡪asynchronous communication  🡪reminder when inactive  CG: waiting list | IG: 69  CG: 36 | A, B, C | A= +  B= -/+  C= + |
| RCT: Randomized controlled trial, IG: Intervention group, CG: Control group, CBT: Cognitive behavioral therapy, PST: Problem-solving therapy, TAU: Treatment as usual. | | | | | | | | |

Table 3: Study characteristics in the area of relapse prevention

| **Study** | **Country** | **Funding** | **Study design** | **Study period** | **Technological approach** | **Sample size** | **Outcomes**  **Depressive symptoms (A)**  **Adherence (B)**  **Quality of life (C)**  **Economic aspects (D)** | **Evidence**  **+ benefit**  **-/+ no benefit**  **~ inconsistent effects** |
| --- | --- | --- | --- | --- | --- | --- | --- | --- |
| Klein et al.  (2018)  [18] | NL | State | RCT | 104 weeks | IG: mobile preventive CBT and minimal therapist support and mood monitoring via text messages  CG: TAU | IG: 132  CG: 132 | D | D= -/+ |
| Kraft et al. (2017)  [14] | GER | State | RCT | 16 weeks | IG: feedback on mindfulness exercises via text messages  IG and CG: introduction to various mindfulness exercises | IG: 21  CG: 20 | B | B= -/+ |
| Lauritsen et al. (2017)  [19] | DEN | Private | Observa-tional study | 4 weeks | Use of a web-based system for entering data on mood, sleep, activity, medication  🡪weekly telephone contact to discuss the data reminder of participants via text messages for data entry | N: 45 | A, B, C | A= +  B= +  C= + |
| Schlicker et al. (2017)  [15] | GER | Not speci-fied | RCT | 10 weeks | IG1: standardized text messages  IG2: individual text messages  🡪short messages sent to maintain the learning units  CG: no text messages | IG 1: 77  IG 2: 73  CG: 76 | A | A= +  (only for IG1)  A= -/+  (only for IG 2) |
| Schma-deke et al.  (2015)  [16] | GER | Private | CCT | 12 weeks | IG: post-inpatient treatment using a telerehabilitation program on a smartphone based on CBT  CG: post-inpatient treatment | IG: 50  CG: 42 | A | A= + |
| Segal et al.  (2020)  [20] | USA | State | RCT | 12 weeks | IG+ TAU: web-based intervention that delivers mindfulness-based CBT, plus usual depression treatment  CG: TAU | IG: 230  CG: 230 | A | A= + |
| Zwerenz et al. (2019)  [17] | GER | State | RCT | 12 weeks | IG: web-based self-help program based on CBT as add- on to multimodal inpatient treatment of depressed patient  🡪reminders via E-Mail or short messages to ensure continuity  CG: TAU and access to an information platform | IG: 108  CG: 107 | A, C | A= +  C= + |
| RCT: Randomized controlled trial, CCT: Clinical controlled trial, IG: Intervention group, CG: Control group, CBT: Cognitive behavioral therapy, TAU: Treatment as usual. | | | | | | | | |

Table 4: Study characteristics in the area of therapy

| **Study** | **Country** | **Funding** | **Study design** | **Study period** | **Technological approach** | **Sample size** | **Outcomes**  **Depressive symptoms (A)**  **Adherence (B)**  **Quality of life (C)**  **Economic aspects (D)** | **Evidence**  **+ benefit**  **-/+ no benefit**  **~ inconsistent effects** |
| --- | --- | --- | --- | --- | --- | --- | --- | --- |
| Aguilera et al. (2017)  [21] | USA | Private and State | Non rando-mized CT | 16 weeks | IG and CG: CBT in group format  IG: additional text messages with therapeutic content, evaluation scales and reminders | IG: 45  CG: 40 | A, B | B = -/+  A = -/+ |
| Agyapong et al. (2017)  [61] | CAN | State | RCT | 12 weeks | IG: received supportive text messages twice a day with therapeutic content and mood improvements  CG: TAU | IG: 35  CG: 38 | A | A= + |
| Bakker et al.  (2018)  [50] | AUS | Not specified | 4- armed RCT | 4 weeks | IG 1: App use with CBT  IG 2: App use with self-monitoring of mood  IG 3: App use with individual strategies for CBT  CG: waiting list | IG 1: 56  IG 2: 56  IG 3: 50  CG: 64 | A | A= +  (in all IG) |
| Beiwinkel et al. (2017)  [39] | GER | EU | RCT | 12 weeks | IG: web-based weekly intervention with perception training and advice giving  CG: unguided web-based psychoeducation | IG: 100  CG: 80 | A, C | A= +  C= -/+ |
| Berger et al.  (2017)  [40] | GER | State | RCT | 12 weeks | IG: regular psychotherapy and access to web-based intervention with psycho-educational information and exercises  CG: weekly psychotherapy | IG: 51  CG: 47 | A, C | A= +  C= -/+ |
| Blackwell et al. (2015)  [67] | UK | Private | RCT | 4 weeks | IG: visual CBT on a website consisting of positive listening exercises and positive word- image stimuli  🡪reminder  CG: positive and negative listening exercises and word-picture stimuli | IG: 76  CG: 74 | A | A = + |
| Bücker et al.  (2019)  [41] | GER | State | RCT | 6 weeks | IG: access to web-based self-help program  🡪based on CBT and techniques of mindfulness  🡪no direct guidance, only support via messaging in case of technical questions  🡪automated reminders via e-mail in case of longer inactivation  CG: waiting list  🡪access to web-based intervention after 6 weeks | IG: 62  CG: 63 | A, B, C | A= -/+  B= -/+  C= -/+ |
| Calkins et al.  (2015)  [22] | USA | Not specified | RCT | 2 weeks | IG: web-based cognitive control training with exercises to strengthen emotional and cognitive functions  CG: visual tasks | IG: 24  CG: 24 | A | A= + |
| Castro et al.  (2018)  [57] | ESP | State | RCT | 52 weeks | IG: web-based self-help intervention with various psychiatric strategies and therapeutic support  CG: web-based self-help intervention without therapeutic support | IG: 96  CG: 98 | B | B= -/+ |
| Clarke et al.  (2015)  [51] | AUS | State | RCT | 8 weeks | IG: fully automated mobile and web-based self-help intervention 🡪diary function  🡪tracking (for selected parameters)  🡪"snippets"= motivational sayings, everyday tips  🡪reminder  CG: waiting list | IG: 231  CG: 489 | A | A= + |
| Crisp et al.  (2014)  [52] | AUS | State | 4- armed RCT | 12 weeks | IG1: web-based intervention IG2: access to internet support group  IG3: combination of depression training and support group  CG: TAU | IG 1: 121  IG 2: 123  IG 3: 117  CG: 117 | C | C= +  (only for IG 3) |
| Dimidjian et al. (2014)  [23] | USA | State | Open and quasi-experi-mental trial | 24 weeks | IG: online- based mindfulness intervention with exercises  Matched-CG: Guideline-compliant TAU with medication and psychotherapy | IG: 100  CG: 100 | A, B | A= +  B= -/+ |
| Ebert et al.  (2018)  [42] | GER | EU | RCT | 28 weeks | IG: web-based intervention based on CBT and PST with tasks, exercises and support from an e-coach  CG: waiting list patients with TAU | IG: 102  CG:102 | A, C | A= +  C= + |
| Flygare et al.  (2020)  [65] | SWE | State | RCT | 8 Weeks | IG: access to web-based program with therapist guidance via messaging service  🡪based on CBT  🡪feedback on assignments  CG: access to web-based program with therapist guidance via messaging service  🡪differences in treatment content and no feedback on assignments | IG: 48  CG: 47 | A, B | A= ~  B= -/+ |
| Fogarty et al.  (2017)  [53] | AUS | Private | Usability Study | 4 weeks | Participants received web-based personalized intervention based on the initial assessment intervention included interactive psychoeducational and self-monitoring exercises  🡪designed exclusively for men | N: 144 | A | A= + |
| Geraedts et al. (2014)  [33] | NL | Private | RCT | 52 weeks | IG: web-based self-help intervention  🡪support and feedback from coaches  CG: TAU | IG: 116  CG: 115 | A, D | A= -/+  D= -/+ |
| Geraedts et al. (2015)  [34] | NL | Private | RCT | 6 weeks | IG: web-based self-help intervention based on PST and CBT  🡪support and feedback from coaches  CG: TAU | IG: 116  CG: 115 | A | A= -/+ |
| Gili et al.  (2020)  [58] | ESP | State | 4-armed  RCT | 8 Weeks | IG: access to web-based program based on various psychological treatment techniques  🡪one face-to-face group session and 4 web-based modules  🡪two automated reminders via mobile phone messages per week for encouraging  🡪in case of inactivity for more than a week automated reminder via e-Mail  🡪continued feedback on modules  IG 1 (HLP+ iTAU): access to web-based program with psychoeducational content for healthy lifestyle  IG 2 (PAPP+ iTAU): access to web-based program with psychological interventions for promotion of positive affect  IG 3 (MP+ iTAU): access to web-based program with brief intervention based on mindfulness  CG (iTAU): improved treatment as usual by GP, who were specially trained for diagnosis and treatment of depressed patients | IG1: 54  IG2: 56  IG3: 54  CG: 57 | A, B, C | A= ~  B= -/+  C= ~  (for all IG) |
| Gräfe et al.  (2019)  [43] | GER | State | RCT | 12 weeks | IG: web-based minimally-  guided self-help program based on CBT additionally to TAU  CG: TAU and waiting list  🡪access to web-based intervention after 12 weeks | IG: 509  CG: 504 | D | D= ~ |
| Gräfe et al.  (2020)  [44] | GER | State | RCT | 12 weeks | IG: web-based minimally-  guided self-help program based on CBT additionally to TAU  CG: TAU and an additional digital brochure with general information on depressive disorders and services for people seeking (self-)help | IG: 1904  CG: 1901 | A, C, D | A=+  C=+  D= ~ |
| Hatcher et al. (2018)  [69] | NZ | State | RCT | 12 weeks | IG: web-based intervention with the support of a coach  CG: TAU with information flyer regarding web- based interventions | IG: 35  CG: 28 | A, C | A= -/+  C= -/+ |
| Hirsch et al.  (2017)  [24] | USA | Private | RCT | 26 weeks | IG: web- based intervention with modules for mood monitoring and relapse prevention  🡪reminder  CG: e-mail notifications with information on mental health and depression | IG: 78  CG: 68 | A | A= + |
| Imamura et al. (2016)  [72] | JAP | Not specified | RCT | 16 weeks | IG: access to information website with psycho-educational information and strategies based on CBT  CG: TAU | IG: 618  CG: 618 | A | A= + |
| Jelinek et al.  (2020)  [45] | GER | Not specified | 3-armed  RCT | 2 weeks | web-based unguided intervention for psychoeducational information  IG (iBA): web-based intervention for behavioral activation  Active CG (iMBI): web-based intervention focused on mindfulness practice  CG: TAU and waiting list  🡪access to web-based program after 4 weeks | IG: 37  Active CG: 32  CG: 35 | A, B, C | A= -/+  B= +  (for IG)  C= -/+ |
| Kelders et al.  (2015)  [35] | NL | State | RCT | 12 weeks | IG: web-based intervention with automated support  CG: web-based intervention with human support | IG: 113  CG: 126 | B | A= -/+  B= -/+ |
| Kenter et al.  (2016)  [36] | NL | Private | RCT | 5 weeks | IG: web-based PST with feedback from a coach  CG: TAU with waiting list and self-help flyer | IG: 136  CG: 133 | A, B, C | A= -/+  B= -/+  C= -/+ |
| Khatri et al.  (2014)  [62] | CAN | State | Pilot study | 13 weeks | IG: web-based intervention through a platform with e-mail components, asynchronous discussion forums and therapy information  CG: TAU | IG: 10  CG: 8 | A, B | A= +  B= + |
| Kivi et al. (2014)  [66] | SWE | State | RCT | 12 weeks | IG: web-based intervention with access to interactive elements such as workbook with mindfulness and acceptance exercises  CG: TAU | IG: 45  CG: 47 | A | A= -/+ |
| Kleiboer et al. (2015)  [37] | NL | Private | 5-armed RCT | 6 weeks | IG 1: web-based intervention without support  IG 2: web-based intervention with support if needed  IG 3: web-based intervention with weekly support  CG 1: no web-based intervention but non-specific chat or support  CG 2: TAU | IG 1: 107  IG 2: 108  IG 3: 106  CG 1:110  CG 2:106 | A | A= +  (only for IG 3) |
| Lambert et al. (2018)  [68] | UK | State | RCT | 8 weeks | IG: web-based intervention with access to evidence-based therapy for physical activity promotion and behavioral activation  🡪reminder  CG: TAU | IG: 32  CG: 30 | A, B | A=+  B=+ |
| Lappa-lainen et al.  (2014)  [63] | FIN | Not specified | RCT | 6 weeks | IG: web-based intervention based on ACT consisting of texts, images and audio-based self-help materials with weekly support and feedback from therapists  CG: ACT by therapists | IG: 19  CG: 19 | A, C | A= +  C= + |
| Lappa-lainen et al.  (2015)  [64] | FIN | Not specified | RCT | 7 weeks | IG: web-based intervention based on ACT consisting of homework and online feedback with email and reminder  CG: TAU | IG: 19  CG: 20 | A | A= + |
| Löbner et al.  (2019)  [46] | GER | State | RCT | 6 weeks | IG+TAU: web-based self-help intervention based on CBT  CG: TAU | IG: 320  CG: 327 | A | A= + |
| Ludtke et al.  (2018)  [47] | GER | Not specified | RCT | 4 weeks | IG: access to a self-help app  with cognitive, mindfulness-based, social skills- based and activation tasks  🡪reminder  CG: TAU | IG: 45  CG: 45 | A, C | A= -/+  C= -/+ |
| Mira et al. (2017)  [59] | ESP | State | 3- armed RCT | 12 weeks | IG 1: web-based intervention based on CBT, psychoeducation and behavioral activation  🡪recording/tracking mood and activity using the rating scale  🡪standardized/ automated text messages to ensure continuity  IG 2: web-based intervention based on CBT, psychoeducation and behavioral activation with weekly telephone contact with therapists  🡪encouragement and motivation of the participants  CG: waiting list  🡪access to web-based intervention after 12 weeks | IG 1: 44  IG 2: 44  CG: 44 | A, B | A= +  B= +  (for all IG) |
| Mira et al. (2018)  [60] | ESP | State | 3- armed RCT | 12 weeks | IG 1: web-based intervention based on positive strategies with automated support  IG 2: web-based intervention based positive strategies with automated support and human help  CG: waiting list  🡪access to web-based intervention after 12 weeks | IG 1: 36  IG 2: 44  CG: 44 | A, B | B = +  A= +  (for all IG) |
| Mohr et al.  (2017)  [25] | USA | State | Pilot study | 8 weeks | app-based intervention  🡪various apps for coping with diseases  🡪recommendations regarding the selection of the app  🡪guidance to ensure continuity and answer questions that arise  🡪at the beginning contact by phone, then weekly text messages | N: 105 | A | A= + |
| Pfeiffer et al.  (2017)  [26] | USA | State | Pilot study | 12 weeks | app-based intervention  🡪therapeutic text messages  🡪rating system for evaluating the day  🡪possibility to actively request help if necessary | N: 190 | A, B | A= +  B= + |
| Pictet et al.  (2015)  [54] | CHE | Not specified | 3- armed RCT | 2 weeks | IG: web-based cognitive restructuring based on "cognitive bias modification" Change in cognitive processes and dysfunctional thoughts  🡪enhancement of visual imagination  CG 1: web-based intervention  🡪imagination process with positive and negative scenarios CG 2: waiting list | IG: 34  CG 1: 34  CG 2: 33 | A | A= +  (for IG) |
| Pots et al. (2016)  [38] | NL | Not specified | 3- armed RCT | 12 weeks | IG: web-based intervention  🡪personalized feedback after the module has been completed CG 1: web-based "writing therapy"  🡪personalized feedback after the writing task has been completed  CG 2: waiting list and TAU  🡪access to web-based intervention after 24 weeks | IG: 182  CG 1: 67  CG 2: 87 | A | A= +  (for IG) |
| Richards et al. (2015)  [71] | IRE | State | RCT | 8 weeks | IG: web-based intervention  🡪feedback to encourage and ensure continuity  CG: waiting list  🡪access to the web-based intervention after 8 weeks | IG: 133  CG: 129 | A | A= + |
| Roepke et al.  (2015)  [27] | USA | Not specified | 3- armed RCT | 4 weeks | IG 1: smartphone-/ web-based intervention for coping with disease and social support via discussion forums and integration of Facebook  IG 2: modified version of the web-based intervention  CG: waiting list and TAU | IG 1: 97  IG 2: 93  CG: 93 | A, C | A= +  C= +  (for all IG) |
| Rosso et al.  (2017)  [28] | USA | State | RCT | 10 weeks | IG: web-based intervention  guided by Avatar  🡪weekly contact by phone  🡪contact psychotherapists only if symptoms worsen and suicidal intentions occur  CG: "monitored attention control", i.e. active CG  🡪access to the web-based application, but no interactive modules  🡪weekly contact by phone | IG: 37  CG: 40 | A | A= + |
| Sandoval et al. (2017)  [29] | USA | State | RCT | 7 weeks | IG: intervention based on virtual therapy  🡪guidance by virtual therapists 🡪feedback function  CG: TAU | IG: 20  CG: 25 | A, B | A= +  B= + |
| Schure et al.  (2019)  [31] | USA | State | Pilot study | 8 weeks | IG: video-based fully automated intervention accessed with a web browser or mobile app and based on CBT  🡪personalized content, exercises, and recommendations/feedback  for participants based on their input and progress  CG: received link to general depression information and waiting list  🡪access to web-based intervention after 8 weeks | IG: 181  CG: 162 | A | A= + |
| Schuster et al. (2018)  [70] | AUT | Not specified | Quasi-experimental trial | 7 weeks | traditional psychoeducation in group format and web-based intervention with personal support | N: 26 | A, B | A= +  B= + |
| Stiles-Shields et al.  (2019)  [31] | USA | State | Pilot Study | 6 weeks | IG 1 (Boost Me): app-based intervention with core strategies based on behavioral activation (BA)  IG 2 (Thought Challenger): app-based intervention with core strategies based on cognitive therapy  🡪publicly available  🡪weekly coaching via  phone or email  🡪all coaching calls were brief (i.e., typically five minutes or less) and were aimed at maintaining engagement with the app, and not in providing therapeutic intervention  CG: waiting list  🡪access to web-based intervention after 6 weeks | IG 1: 10  IG 2: 10  CG: 10 | A | A= ~  (for all IG) |
| Tomasino et al. (2017)  [32] | USA | State | 3- armed pilot study | 8 weeks | IG 1: web-based intervention  🡪individual support for motivation via telephone/ text messages  IG 2: web-based intervention and additional use of interactive elements to build social relationships with other participants (comments, likes, etc.)  🡪individual support for motivation via telephone/ text messages  CG: waiting list | IG 1: 12  IG 2: 23  CG: 12 | A, B | A= +  (for all IG) |
| Wagner et al. (2014)  [55] | CHE | Private | RCT | 8 weeks | IG: web-based intervention and therapeutic support during the intervention with individual feedback/support for the writing tasks  CG: TAU | IG: 32  CG: 30 | A | A= + |
| Wahle et al.  (2016)  [56] | CHE | Private | Pilot study | 36 weeks | Mobile sensing and support (MOSS) app  🡪app-based intervention  🡪context information about participants is collected and evaluated using sensors, e.g. pedometer, GPS, number of calls etc.  🡪recommendations are provided in accordance with context and everyday conditions | N: 126 | A | A= + |
| Zagor-scak et al. (2019)  [48] | GER | Private | RCT | 6 weeks | IG: web-based intervention based on CBT with feedback  individualized by a counselor  CG: web-based intervention based on CBT with receiving standardized feedback  🡪feedback was offered via  written messages within a  password-protected Internet  platform after the completion  of each module  🡪clients in both intervention groups could receive contact on demand in case of technical problems or specific questions concerning the intervention | IG: 555  CG: 534 | A | A= -/+ |
| Zwerenz et al. (2017)  [49] | GER | Private | RCT | 12 weeks | IG: traditional/ inpatient psychotherapy and access to the web-based intervention  🡪dialog-like self-help intervention  🡪standardized reminders to ensure continuity  🡪access to web-based intervention even after discharge from inpatient treatment  CG: TAU and access to an online information platform | IG: 115  CG: 114 | A, C, D | A= +  C= +  D= -/+ |
| RCT: Randomized controlled trial, CT: Controlled trial, IG: Intervention group, CG: Control group, TAU: Treatment as Usual, CBT: Cognitive Behavior Therapy, PST: Problem solving therapy, BA: Behavioral activation, GP: General practitioners. | | | | | | | | |
